# Optimizing left atrial appendage imaging: the diagnostic value of left lateral decubitus cardiac CT angiography

**DOI:** 10.3389/fcvm.2026.1725901

**Published:** 2026-06-05

**Authors:** Zihao Wang, ChenJing Wu, Yi Mang, Zhuang Zhuang, Xinshi Huang, Zhenzhang Wang

**Affiliations:** 1 Department of Radiology, The First Affiliated Hospital of Wenzhou Medical University, Wenzhou, China; 2 Department of Cardiovascular, The First Affiliated Hospital of Ningbo University, Ningbo, China; 3 Department of Ultrasound, The First Affiliated Hospital of Wenzhou Medical University, Wenzhou, China

**Keywords:** atrial fibrillation, computed tomography angiography, left atrial appendage, left atrial appendage thrombus, leftlateral decubitus position

## Abstract

**Background:**

Cardiac computed tomography angiography (CCTA) is widely used to detect left atrial appendage (LAA) thrombus before catheter ablation; however, the conventional biphasic supine (BS) CCTA doubles radiation exposure. This study evaluated whether single-phase left lateral decubitus (LLD) CCTA can maintain diagnostic accuracy while reducing radiation dose.

**Methods:**

We retrospectively included 114 patients with atrial fibrillation who underwent CCTA between December 2022 and December 2024 (BS group, *n* = 57; LLD group, *n* = 57). Transesophageal echocardiography served as the reference standard. Two experienced radiologists independently reviewed each phase of the CCTA images for thrombus detection. We assessed the distribution pattern of contrast enhancement in the LAA during the arterial phase in the supine position and compared LAA filling, diagnostic performance, and effective radiation dose between the BS and LLD protocols.

**Result:**

In the early arterial phase of the BS group, contrast attenuation in the LAA progressively decreased from the base toward the apex. The LLD position significantly improved contrast agent filling in the left atrial appendage apex (median 444.8 HU vs. 70.3 HU, Z = 8.10, *p* < 0.001) and reduced the early filling defect rate from 28% to 8.8% (*p* < 0.001) compared to the arterial phase in the BS group. TEE confirmed LAA thrombus in 13 patients (11%). For LLD-CCTA, sensitivity was 100% (95% CI, 61–100), specificity was 0.94 (95% CI, 0.87–0.97), PPV was 0.70 (95% CI, 0.40–0.89), and NPV was 1.00 (95% CI, 0.92–1.00)—performance that was not statistically inferior to the biphasic protocol (*p* = 0.35). The mean effective dose was nearly halved, from 7.84 ± 2.44 mSv to 3.89 ± 1.26 mSv (*p* < 0.001).

**Conclusion:**

Single-phase LLD-CCTA maintains diagnostic accuracy while reducing radiation exposure by approximately 50% and streamlining workflow. These advantages support prospective, multicenter validation before considering routine clinical adoption.

## 1 Introduction

Atrial fibrillation (AF) is one of the most common arrhythmias and accounts for approximately one-third of cardioembolic strokes, with up to 90% of thrombi originating in the left atrial appendage (LAA) (1, 2). Transesophageal echocardiography (TEE) remains the reference standard for detecting LAA thrombi; however, it is semi-invasive, time-consuming, and its diagnostic performance is highly dependent on operator expertise (3). Over the past decade, cardiac computed tomography angiography (CCTA) has emerged as a reliable, non-invasive alternative to TEE. A large meta-analysis reported a pooled sensitivity of 0.95 and specificity of 0.89, comparable to TEE, with the added advantages of operator independence and clear visualization of pulmonary vein anatomy (4). The 2023 SCAI/HRS expert consensus states that CCTA can be used as a routine diagnostic tool before and after left atrial appendage occlusion surgery, and points out that its priority is higher than TEE in postoperative follow-up (5). The current LAA CCTA workflow follows an expert recommendation document that advocates a biphasic supine approach with early arterial collection followed by a delayed scan of 60 to 180 s to rule out a pseudo-filling defect caused by slow blood flow in the left atrial appendage (6). Although this method increases specificity to over 95%, it prolongs the scanning time and increases the radiation dose by approximately 3–4 mSv (7). Alternative imaging strategies that can maintain diagnostic accuracy while improving opacification and reducing dose therefore merit further investigation.

Earlier work has explored posture to improve LAA opacification, but the evidence remains fragmentary. Several studies suggest that the prone position can improve the accuracy of CCTA in diagnosing LAA thrombus and propose that gravity may affect the distribution of contrast agent within the left atrial appendage (8, 9). In a fluid dynamics study (10), it was noted that differences in prone position may lead to altered hemodynamics in the left atrium. This inspired us to consider whether placing the left atrial appendage at the lowest position in the body during the arterial period could improve its early pseudo-defect. Therefore, we proposed the hypothesis that placing the apex of the left atrial appendage at the lowest point of the body during the early arterial phase can improve early arterial imaging results of the left atrial appendage.

Building on this background, we conducted a study to evaluate early-phase LAA CCTA in the left lateral decubitus position. Specifically, we characterized the arterial-phase distribution of contrast within the LAA, compared LAA opacification and diagnostic performance between the LLD and conventional supine protocols, and assessed the impact of positioning on radiation dose.

## 2 Method

### 2.1 Patient population

**Figure 1:**
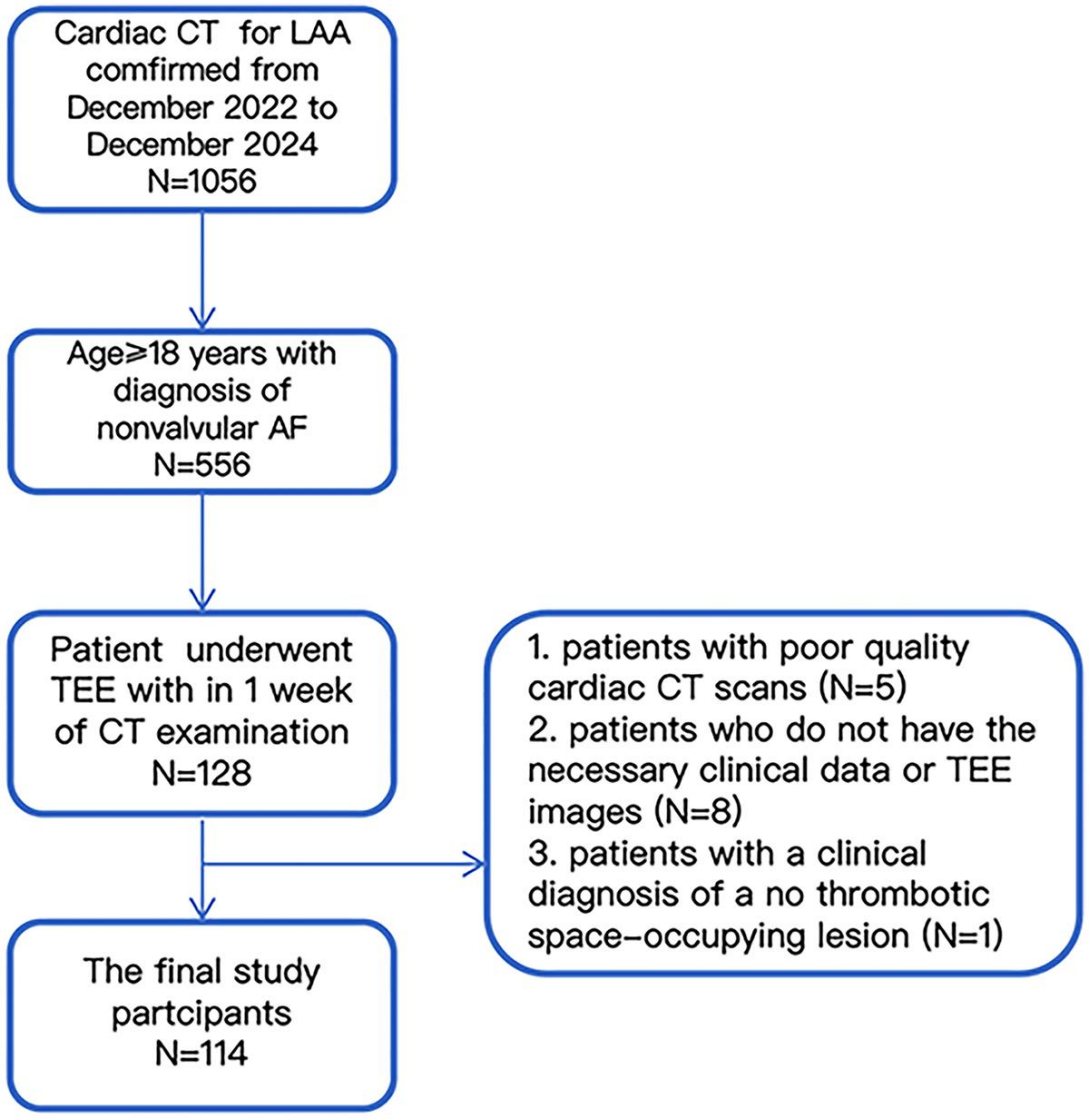
Patient enrollment flowchart.

The study was approved by the Institutional Ethics Committee and was conducted in accordance with the Declaration of Helsinki. All patients provided written informed consent. This was a single-center retrospective study that included patients with nonvalvular AF from December 2022 to December 2024. All patients underwent TEE within 48 h after CCTA. The inclusion and exclusion criteria for patients were as follows: Inclusion criteria 1. age ≥ 18 years, 2. patients with a definite diagnosis of nonvalvular AF, 3. patients who had signed an informed consent for admission to the hospital and complied with the ethical requirements and exclusion criteria: (1). patients with poor quality cardiac CT scans, i.e., images that do not meet diagnostic requirements (expected to be caused by severe artifacts) or left atrial enhancement CT values of less than 200 Hounsfield units (HU) as judged by 2 qualified senior radiologists; (2). patients who do not have the necessary clinical data or TEE images; and (3). patients with a clinical diagnosis of a no thrombotic space-occupying lesion, such as a left atrial tumor. Figure 1 is the research flowchart, showing detailed information on enrollment and exclusion.

### 2.2 Transesophageal and transthoracic echocardiography

Transesophageal echocardiography was performed using a Philips iE33 (Philips Medical Systems, USA) system to evaluate the left atrium and ventricle. The operating frequency of the probe was 5.5–6 MHz. Multiplanar scanning was performed when the probe was located 35 cm inside the esophagus. The final determination of a blood clot is made by an experienced cardiac sonographer. Transthoracic echocardiography was performed using a Philips EPIQ7 (Philips Medical Systems, USA) to obtain LV volumes, ejection fraction (LVEF), left atrial (LA) diameter, etc.

### 2.3 CT acquisition protocols

#### 2.3.1 Biphasic supine position CCTA

CCTA was performed with GE Apex sensing 256-slice spiral CT. No beta-blockers were used for regulation of heart rate in any of the enrollees. The imaging protocol includes standard arterial phase acquisition for assessment of left atrial and pulmonary vein anatomy, followed by a delayed phase scan 1 min later to assess left atrial appendage visualization. During the scanning, the patient was placed in the supine position, a high-voltage injector and electrode leads were connected, and the scan was gated using a prospective electrocardiogram (ECG). The prospective gated center is located at 30%–80% of the RR interval, ranging from the aortic arch to the bottom of the heart. The tube voltage and current were adapted to the patient's body mass index (BMI) (100 kVp for patients with BMI ≤ 30 kg/m^2^ and 120 kVp for patients with BMI > 30 kg/m^2^). All scans are equipped with automatic tube current modulation technology. All examinations are performed using scanners equipped with automatic tube current modulation. Image reconstruction is performed using a deep learning-based algorithm to improve the signal-to-noise ratio. The gantry rotation time was 0.35 s, and the image was reconstructed with a slice thickness of 0.6 mm. The contrast medium was iopromide with a 350 mgI/mL concentration. This volume was calculated based on the patient's weight, 1 mL of contrast media per 1 kg of body weight. The contrast media was injected into the cubital IV cannula at a rate of 5 mL/s, followed by a 70 mL bolus of saline solution. The region of interest was placed in the ascending aorta. The scanning length, time, and dose-length product (DLP) of CT are recorded from the scanner console, and the DLP is multiplied by the previously recommended conversion factor (0.014 mSv·cm/mGy) to calculate the effective radiation dose at different stages. All scans were performed by experienced technicians who have been engaged in cardiac CT scanning for more than 3 years and were carried out in strict accordance with the operation specifications.

#### 2.3.2 Single-phase left lateral decubitus position CCTA

In the single-phase left lateral wall (LLD) CCTA protocol, the region of interest is placed in the left atrial appendage, and image acquisition is triggered 6 s after attenuation reaches 200 HU. During the scan, the patient maintains the left position, rotates 90°, hands on the head, and legs bent to 120° to stabilize the lateral trunk position. All scanning parameters are the same as biphasic supine position (BS) CCTA.

### 2.4 Analysis of the computed tomography image

**Figure 2:**
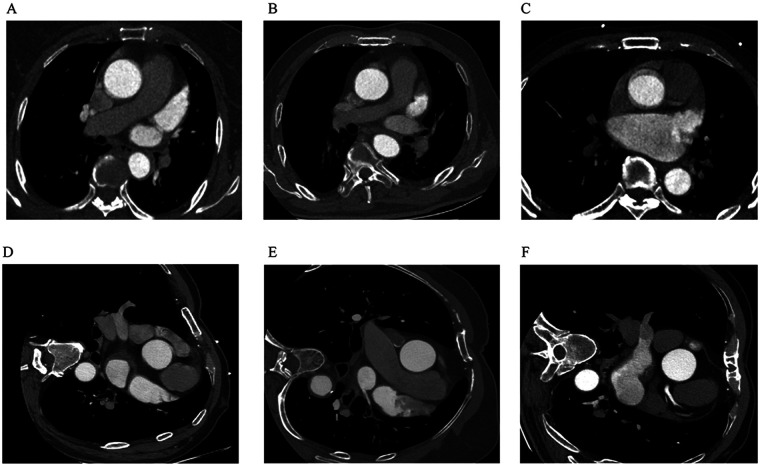
**(A–F)** Show several filling patterns of the LAA in the supine position and left lateral position, respectively, in the order of normal filling (complete filling of LAA contrast), poor filling (any filling defect ≥ 100 HU), or thrombus (filling defect < 100 HU).

**Figure 3:**
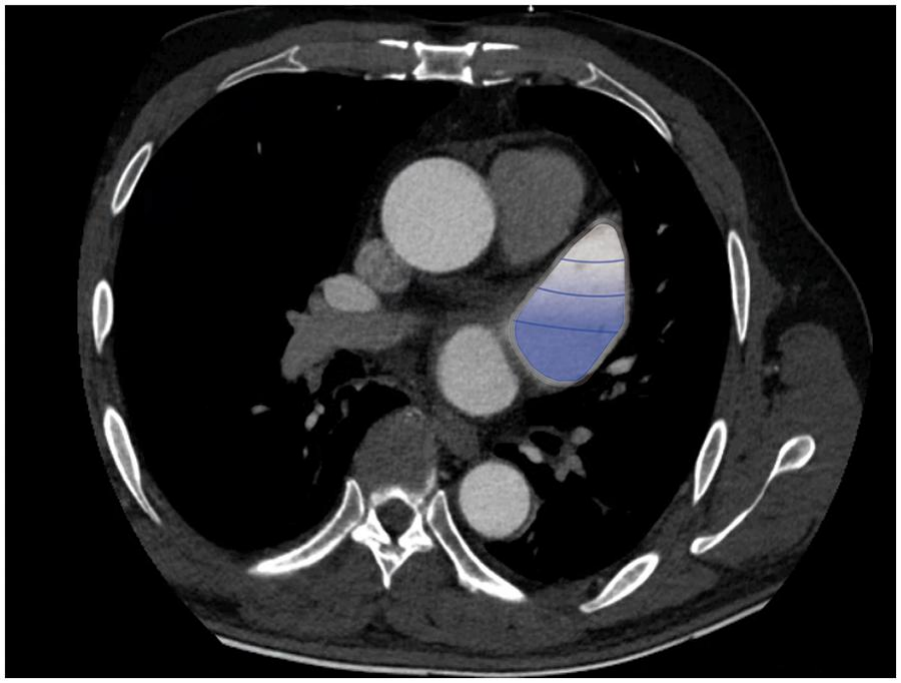
Schematic diagram of the left atrial appendage divided into four equal sections from the apex to the base.

All CT images were interpreted by two radiologists (Z.H.W., with 5 years of experience in cardiovascular imaging; Y.M., with 7 years of experience), both of whom were blinded to the clinical data and each other's assessments. Discrepancies were resolved by consensus after joint review. CT images of the left atrial appendage (LAA) are classified into three categories based on contrast enhancement: normal filling (homogeneous enhancement), poor filling (attenuation defect ≥ 100 HU), and thrombus (attenuation < 100 HU). Representative examples are shown in Figure 2 (11, 12). To further analyze the early distribution of contrast agent in the left atrial appendage, we divided the left atrial appendage into four equal parts from the apex to the base (apex, neck, body, base). Radiologists independently placed 3 mm circular regions of interest (ROIs) at four predefined axial levels (apex, middle, mid-lower, and base, shown in Figure 3). Each ROI was placed in the center of the LAA lumen to minimize trabecular interference, and the mean attenuation was calculated from three consecutive axial slices. If the difference between slices exceeded 10 HU, the consistency of the measurements was reassessed.

### 2.5 Statistical analysis

Statistical analysis was carried out with SPSS version 30.0 (IBM Corp., Armonk, NY, USA) and R version 4.5.1 (R Foundation for Statistical Computing, 2018, Vienna, Austria). The Shapiro–Wilk test was performed for each continuous variable to assess normality. Data conforming to normal distribution are presented as mean ± standard deviation (SD), and data not normally distributed are presented as median (Q1, Q3). A chi-square test (or Fisher's exact test when the expected number of cells is less than 5) was used to compare categorical variables. To verify the within-scan apex-to-base contrast gradient in the single-phase supine acquisition, a linear mixed-effects model with patient as a random intercept was fitted. The Wilcoxon signed rank test was used to compare vertex HU values for paired scans within the biphasic group, and the Mann–Whitney *U*-test was used for comparisons between the biphasic and left-sided groups. Using transesophageal echocardiography as the reference standard, 2 × 2 contingency tables were constructed for each CT protocol. Sensitivity, specificity, positive predictive value (PPV), negative predictive value (NPV), and Cohen's κ were calculated with Wilson 95% confidence intervals. Because each protocol generated a single binary decision point, the ROC curve degenerated to a two-segment line; the resulting single-threshold AUC was therefore defined analytically as AUC^∗^ = (Sensitivity + Specificity)/2. Differences in AUC* between the two independent cohorts were tested with a large-sample *z*-test derived from binomial variance and verified by a 10,000-sample non-parametric bootstrap. All tests were two-sided, and *p* < 0.05 was considered significant.

## 3 Result

### 3.1 Patient characteristics

A total of 114 participants were included, with 57 in the BS group and 57 in the LLD group. The median age was slightly higher in the BS group [70 [63–76] vs. 68 [62–74] years], and a higher proportion of female patients was observed (40.4% vs. 29.8%) (*p* > 0.05). Comorbidities, atrial fibrillation type, and anticoagulation strategies were similarly distributed. Risk scores, including CHA₂DS₂-VASc and HAS-BLED, as well as echocardiographic parameters such as LVEF and LA diameter, showed no meaningful differences between groups (*p* > 0.05). Regarding LAA morphology, the distribution of types (chicken wing, cactus, cauliflower, windsock) was not significantly different (*p* > 0.05). The incidence of poor LAA contrast filling on first-phase CT was higher in the BS group compared to the LLD group (28.1% vs. 8.8%) (*p* = 0.01). CT-suspected thrombus was identified in 21.1% of the BS group and 17.5% of the LLD group. Details of the baseline were shown in Table 1.

**Table 1:** Study characteristics of participant.

Characteristic	Category	Biphasic supine position (*n* = 57)	Left lateral decubitus position (*n* = 57)	*P*-value
Age		70 [63–76]	68 [62–74]	0.638
Sex (%)	Female	23 (40.4)	17 (29.8)	0.326
	Male	34 (59.6)	40 (70.2)	
BMI		24.63 ± 3.59	24.56 ± 3.40	0.918
AF type (%)	Paroxysmal	33 (57.9)	30 (52.6)	0.706
	Persistence	24 (42.1)	27 (47.4)	
Hypertension (yes, %)		31 (54.4)	34 (59.6)	0.705
Diabetes (yes, %)		15 (26.3)	17 (29.8)	0.835
Coronary Heart Disease (yes, %)		13 (22.8)	9 (15.8)	0.476
Stroke (yes, %)		12 (21.1)	17 (29.8)	0.390
Heart failure (yes, %)		11 (19.3)	12 (21.1)	1.000
Anticoagulant (%)	VKA	1 (1.8)	2 (3.5)	1.000
	NOAC	56 (98.2)	55 (96.5)	
CHA_2_DS_2_-VASc		3.00 [2.00, 4.00]	3.00 [2.00, 4.00]	0.485
HAS-BLED score		2.00 [1.00, 3.00]	2.00 [1.00, 3.00]	0.135
INR		1.27 ± 0.28	1.18 ± 0.18	0.051
LVEF		61.87 ± 7.77	61.73 ± 10.30	0.937
LA transverse diameter		77.46 ± 9.03	75.39 ± 8.48	0.210
LA anteroposterior diameter		46.84 ± 6.94	47.30 ± 8.36	0.752
LAA type (%)	Chicken Wings	24 (42.1)	16 (28.1)	0.222
	Cactus	27 (47.4)	28 (49.1)	
	Cauliflower	3 (5.3)	8 (14.0)	
	Windsock	3 (5.3)	5 (8.8)	
CT imaging features				
CT poor filling (%)	Arterial Phase	16 (28.1)	5 (8.8)	0.01
	Delayed Phase	6 (10.5)		
CT thrombus (%)		12 (21.1)	10 (17.5)	0.19

Data are mean ± SD, *n* (%), or median. BMI, body mass index; INR, international normalized ratio; LVEF, left ventricular ejection fraction; LA, left atrium; PT, prothrombin time; TTE, transthoracic echocardiography, TIA, transient ischemic attack.

### 3.2 Apex-to-base enhancement gradient in supine position

In the arterial phase of the BS group, the contrast agent density of the LAA was observed to gradually decrease from the base point toward the apex. The linear mixed-effects model demonstrated that each anatomical step from apex to base was associated with an average increase of 96.7 Hounsfield Units (β = 96.70, 95% CI: 88.18–105.22, *p* < 0.001). Likelihood ratio testing further supported the statistical significance of this gradient (*χ*^2^ = 62.17, *p* < 0.001).

### 3.3 Effect of delayed phase and left lateral decubitus position on LAA filling

The delayed phase significantly improved contrast opacification at the LAA apex within the BS group compared to the early phase [70.3[41.96–132.33] to 190.0 [84.25–304.6], *Z* = 5.51, *p* < 0.001]. Notably, the CT values at the LAA apex in the LLD group were higher than those in the BS group, both in the early arterial phase (median 444.8 vs. 70.3 HU, Z = 8.10, *p* < 0.001) and the delayed phase (median 444.8 vs. 190.0 HU, *Z* = 5.78, *p* < 0.001). Moreover, in terms of poor filling, only 5 patients (8.8%) in the LLD group were markedly lower compared to 16 patients (28.1%) in the early supine position (*p* = 0.010), slightly lower in the delayed phase (10.5%), although the latter difference did not reach statistical significance (*p* = 0.739).

### 3.4 Diagnostic performance of CT against TEE

A total of 114 patients were analyzed, with CT-based LAA thrombus evaluation compared against TEE as the reference standard. In the BS group, CT identified thrombus in 12 patients, of whom 6 (50.0%) were confirmed by TEE. In the LLD group, CT identified thrombus in 10 patients, with 7 (70.0%) confirmed by TEE. In both examination methods, sensitivity and negative NPV are 100%, indicating that CT scans did not miss any thrombi detected by TEE. However, specificity differed between groups, being 88% in the BS group and 94% in the LLD group, while PPV was 50% and 70%, respectively. And the LLD group also a better agreement (κ = 0.794) than the BS group (κ = 0.612). The AUC was 0.94 for the BS group and 0.97 for the LLD group. Although the LLD group showed numerically higher AUC values, a large-sample *Z*-test revealed no statistically significant difference in diagnostic performance between the two protocols (*P* = 0.35). The detailed diagnostic performance metrics are summarized in Table 2.

**Table 2:** Diagnostic performance of cardiac CT against TEE.

Group	AUC	Accuracy	Sensitivity	Specificity	PPV	NPV	Kappa
BS group	0.941	0.89 (0.79–0.95)	1.00 (0.61–1.00)	0.88 (0.77–0.94)	0.50 (0.25–0.75)	1.00 (0.92–1.00)	0.612
LLD group	0.970	0.95 (0.86–0.98)	1.00 (0.65–1.00)	0.94 (0.84–0.98)	0.70 (0.40–0.89)	1.00 (0.92–1.00)	0.794

Data are expressed as value (%) and 95% confidence interval.

### 3.5 The radiation dose between two groups

Radiation dose was evaluated by estimating the effective dose, calculated as the product of the DLP and a standard conversion factor for chest CT (k = 0.014 mSv·cm/mGy). The mean effective dose was significantly higher in the BS group (7.84 ± 2.44 mSv) compared to the LLD group (3.89 ± 1.26 mSv) (*t* = 4.38, *p* < 0.01). These findings indicate that the LLD protocol significantly reduces radiation exposure by nearly 50% compared to the BS group. Radiation dose parameters are summarized in Table 3.

**Table 3:** The radiation dose between biphasic supine position and left lateral decubitus position

Group	CTDIvol (mGy)	DLP (mGy·cm)	Effective dose (mSv)	T	*P*
BS group	26.7 ± 5.4	560 ± 174	7.84 ± 2.44		
LLD group	13.5 ± 3.6	278 ± 94	3.89 ± 1.26	4.38	<0.01

DLP, dose-length product; CTDIvol, computed tomography dose index volume

## 4 Discussion

In this study, left-lateral decubitus (LLD) cardiac CT angiography markedly improved contrast distribution within the left atrial appendage (LAA), with attenuation at the apex increasing more than sixfold compared to the conventional biphasic supine (BS) protocol. This enhancement resulted in a significant reduction in distal poor filling (8.8% vs. 28.1%). When referenced against TEE, the LLD protocol achieved 100% sensitivity and negative predictive value. Additionally, radiation exposure was reduced by nearly half, offering a practical and low-dose alternative to the current biphasic standard.

Cardiac CT has become the primary imaging method for excluding thrombi before catheter ablation or left atrial appendage closure. Currently, the clinical practice standard is the biphasic supine scan, which involves a second scan performed 60–180 s after the arterial phase imaging. This has been validated by large-scale meta-analyses, showing its diagnostic performance (sensitivity 0.98, specificity 1.00) to be highly consistent with TEE (4). However, this accuracy comes at the cost of significantly increased radiation exposure. Studies have shown that each additional delayed scan increases the effective dose by approximately 1.5–3 mSv (7, 13). Although the radiation dose for coronary CTA has been reduced by 80% over the past decade, the dual-phase LAA assessment protocol has not benefited accordingly, becoming a bottleneck in current dose control (13). Some research groups have optimized the scanning protocol or improved the contrast agent injection protocol. In a double contrast, single-phase CTA study, this method was found to reduce radiation dose and effectively improve left atrial appendage filling (26/579), but its positive predictive value was only 7.7% (14). In an earlier low-dose dual-phase delayed study, the average dose was 6.7 mSv, which was also significantly higher than our 3.9 mSv in the left lateral decubitus position (15). A recent study using limited scanning of the left atrial appendage in the delayed phase effectively reduced the radiation dose of the dual-phase protocol (mean 3.5 mSv), but the limited scanning operation in the delayed phase increased the time cost of the scan (16). Despite such an ingenious approach, up to a quarter of supine arterial images still show distal low density, further driving the need for more physiological solutions.

Therefore, researchers began to intervene in posture. Several case-control studies and case reports on prone re-scanning have indicated that left atrial appendage filling defects detected in the prone position may disappear in the supine position (17, 18). Nakamura et al. (8) prospectively studied 300 patients with persistent or long-standing AF using a two-phase protocol. Distal filling defects were observed in 40 cases during the early phase, with only 6 remaining in the delayed phase. While the NPV for LAA thrombus reached 100% and sensitivity was 0.9, the delayed scan added ∼1.6 mSv of radiation and extended the exam by about 1 min. In addition, prolonged supine imaging may reduce patient comfort. Recent efforts have shifted from prone positioning to placing patients in the left lateral decubitus position, which lowers the LAA relative to gravity and is more acceptable to patients. Kaliyev et al. (19) conducted a retrospective analysis of 101 patients with atrial fibrillation who underwent single-phase LLD scanning prior to ablation, demonstrating high concordance with TEE (PPV = 100%, NPV = 100%). Compared with previous study focusing on LLD study, we further investigated the distribution pattern of contrast enhancement from the base to the apex of the LAA during the arterial phase in the conventional supine position. We also directly compared the LLD and BS to further evaluate their diagnostic performance, contrast filling characteristics, and radiation exposure, because understanding how patient positioning affects both intracardiac contrast distribution and cumulative dose is crucial for developing safer and more efficient pre-ablation imaging strategies.

Research on the computational fluid dynamics (CFD) of the left atrial appendage provides a potential explanation for the improved imaging quality resulting from positional changes. A CFD work shows different pulmonary venous blood flow distributions (60/40% and 55/45% through the right and left pulmonary veins, respectively) are associated with left atrial appendage blood stasis (20). In addition, gravity and changes in body position can influence this condition, with 59% and 41% of cases occurring in the left lateral position and 37% and 63% in the right lateral position, respectively (21). In a nine-subject AF model, it was noted that left lateral recumbency may increase Left Pulmonary Vein flow by up to 59%, push the main vortex toward the lateral side of the LAA, and reduce Stagnant Blood Volume (∼20%–40%), thereby facilitating LAA flushing (10). These data provide a mechanistic substrate for the marked reduction in distal filling defects seen on prone and LLD cardiac-CT images.

Although the LLD may help early contrast filling and visualization of the left atrial appendage, it does not eliminate the inherent physiological characteristic of slow blood flow, nor can it fully compensate for interpatient anatomical and hemodynamic differences. Therefore, reliance on gravity alone is insufficient to exclude pseudo-filling defects in all cases. Importantly, the clinical value of left lateral imaging lies in its ability to improve early LAA filling in some patients, thereby reducing the need for unnecessary delayed imaging. However, as shown in Figure 2E, persistent or indeterminate filling defects may still be observed, and when diagnostic uncertainty remains, delayed imaging or transesophageal echocardiography is still required.

Several limitations of this analysis should be acknowledged. First, it was a retrospective study conducted at a single tertiary-care centre, which may introduce referral and selection bias. Second, patients with left-atrial attenuation below 200 HU were excluded *a priori*, limiting the generalizability of the findings to low-flow or low-contrast states. Third, the small number of TEE-confirmed thrombi resulted in wide confidence intervals for specificity and PPV, indicating limited statistical power for thrombus confirmation and the need for validation in larger prospective cohorts. In addition, a relatively wide prospective ECG-gated acquisition window was used to accommodate RR interval variability in patients with atrial fibrillation, which may result in image acquisition at different cardiac phases across individuals. Fourth, contrast volume and timing were fixed rather than tailored to patient body habitus or cardiac output. Fifth, inter-reader agreement for region-of-interest placement was not formally assessed, precluding evaluation of reproducibility. Sixth, radiation exposure was expressed as effective dose estimated from DLP conversion factors, without organ-specific dosimetry. Finally, advanced radiation dose–reduction techniques were not incorporated in the present study, which may have influenced the magnitude of the observed radiation dose difference between protocols.

Future prospective, multicenter studies incorporating low-contrast states, individualized contrast protocols, advanced CT techniques, and reproducibility assessment are warranted to validate and extend the applicability of the LLD-CCTA approach.

## 5 Conclusion

Single-phase LLD CCTA offers a pragmatic, patient-friendly route to exclude LAA thrombus, cutting dose by half and reducing the need for delayed imaging while maintaining the negative predictive power required for safe ablation or occlusion workflows.

## Data Availability

The raw data supporting the conclusions of this article will be made available by the authors, without undue reservation.
